# Effect of Site Level Environmental Variables, Spatial Autocorrelation and Sampling Intensity on Arthropod Communities in an Ancient Temperate Lowland Woodland Area

**DOI:** 10.1371/journal.pone.0081541

**Published:** 2013-12-09

**Authors:** Jakub Horak

**Affiliations:** Department of Forest Protection and Entomology, Faculty of Forestry and Wood Sciences, Czech University of Life Sciences Prague, Prague, Czech Republic; University of Innsbruck, Austria

## Abstract

The interaction of arthropods with the environment and the management of their populations is a focus of the ecological agenda. Spatial autocorrelation and under-sampling may generate bias and, when they are ignored, it is hard to determine if results can in any way be trusted. Arthropod communities were studied during two seasons and using two methods: window and panel traps, in an area of ancient temperate lowland woodland of Zebracka (Czech Republic). The composition of arthropod communities was studied focusing on four site level variables (canopy openness, diameter in the breast height and height of tree, and water distance) and finally analysed using two approaches: with and without effects of spatial autocorrelation. I found that the proportion of variance explained by space cannot be ignored (≈20% in both years). Potential bias in analyses of the response of arthropods to site level variables without including spatial co-variables is well illustrated by redundancy analyses. Inclusion of space led to more accurate results, as water distance and tree diameter were significant, showing approximately the same ratio of explained variance and direction in both seasons. Results without spatial co-variables were much more disordered and were difficult to explain. This study showed that neglecting the effects of spatial autocorrelation could lead to wrong conclusions in site level studies and, furthermore, that inclusion of space may lead to more accurate and unambiguous outcomes. Rarefactions showed that lower sampling intensity, when appropriately designed, can produce sufficient results without exploitation of the environment.

## Introduction

Research on diversity of arthropods, management of populations and their interaction with the environment is one of the main topics of the present ecological agenda, especially with respect to potential biotical and abiotic threats [1]. However, recent research on biota suffers from many pitfalls [2], which may lead to biased conclusions [3].

Arthropods are, mostly quickly, responding to environmental changes [1] and knowledge of their response to habitat parameters continues to increase [4], [5]. Many arthropods are influenced by the fact that they are often dispersal-limited and thus not able to reach more distant habitats [6], [7]. Response of arthropods to the environmental variables may be biased by spatial structure of their distribution [8].

With respect to data analyses, spatial autocorrelation is one of the statistical problems encountered when modelling species-environmental relationships [9], [10]. Spatial heterogeneity is defined as either the variation in space in the distribution of a point pattern, or as variation in the qualitative or quantitative value of a surface pattern [11], [12], which can be caused also by site level factors [13]. Spatial dependency within geographic space leads to the spatial autocorrelation [14]. It is known that all of nature is autocorrelated that everything is related to everything else and that objects close to each other are more related than those that are further apart [15] – thus, spatial autocorrelation generates possible bias [16]. Although the existence of spatial autocorrelation does not in itself constitute real bias, it does in terms of what ecologists want to understand [9]. Spatial autocorrelation can be fundamental to building a spatial component into statistical models [17]. If spatial autocorrelation is ignored it is always hard to determine if results can in any way be trusted [3].

Present studies indicate that potential bias of spatial autocorrelation is not absolutely dependent on scale of trapping designs [8], [9], [17], [18]. Thus, mass trapping is not necessary for site level type of studies. Lower trapping intensity in appropriately designed studies gain useful results and may lead to a higher significance without exploitation of the environment [19].

The main goals of this study were to determine the response of arthropod communities to environmental variables at the site level in an area of continuous lowland woodland and to determine how their response could be influenced by sampling intensity and spatial autocorrelation, while employing commonly used trapping methods at the same trees during two seasons.

## Materials and Methods

### Ethics Statement

All necessary permits were obtained for the described study. As the study area was a part of the protected area, this study was undertaken with the permission of the Czech Government (no. 1473/09).

### Study Area

**Figure 1:**
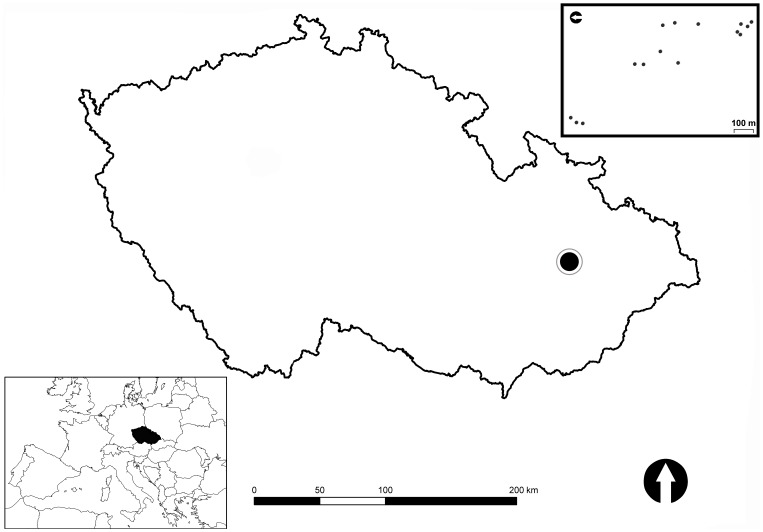
Map of the study sites in Zebracka (Czech Republic).

All sample occasions were situated within approximately 50 ha situated in the national nature reserve of Zebracka (Prerov, Olomouc Region, Central Moravia, Czech Republic; Fig. 1), one of the most continuous (i.e. ancient) deciduous woodlands near the River Becva [20], known from at least the 1750s (i.e. the time of the first Austro-Hungarian Military Mapping). Zebracka is known to be one of the species rich woodlands in Moravia [21].

### Sampling Methods

During the 2009 season, fifteen crossed-panel window traps [22] were used, replaced in 2010 by panel traps [23] – two commonly used methods. All traps were placed on the stems of standing veteran poplars (*Populus*) at 1.3 m. The sampling methods are described in Table 1. All trapped arthropods were identified to the taxon level listed in Table 2.

**Table 1:** Description and comparison of trapping methods used in deciduous woodland area of Zebracka.

Character	Window trap	Panel trap
Season	2009	2010
Number	15	15
Intercept area (m^2^)	0.800	0.063
Colour	Blank	Yellow
Activity	Passive	Active
Medium	Water+NaCl+detergent [22]	Soveurode® Witasek [23]
Selection	Not known	Larger (>1.5 cm) individuals
Trapping activity	20.V.–12.IX.	9.IV.–11.IX.
Trapping days per trap	115	155
Placement	Trunk	Trunk
Height of the centre (m)	1.3	1.3
Irradiance	South	South

**Table 2:** Results of trapped taxa (sorted in alphabetical order) and abundances during the study seasons 2009 and 2010 in a deciduous woodland area of Zebracka.

Taxa	2009	2010	Total
Araneae	383	184	567
Coleoptera	1,756	2,256	4,012
Collembola	15	–	15
Dermaptera	1,090	1	1,091
Diptera	546	9,485	10,031
Ephemeroptera	9	2	11
Glomerida	104	–	104
Hemiptera: Heteroptera	95	–	95
Hemiptera: Sternorrhyncha	106	19,019	19,125
Hymenoptera	2,413	1,269	3,682
Chilopoda	3	–	3
Isopoda	41	–	41
Ixodida	20	–	20
Julida	182	6	188
Lepidoptera	284	74	358
Mecoptera	10	12	22
Neuroptera	–	11	11
Opilionida	84	4	88
Orthoptera	37	–	37
Prostigmata	5	–	5
Pseudoscorpionida	6	2	8
Pulmonata	76	–	76
Raphidioptera	14	–	14
Trichoptera	39	1	40

### Study Variables

I focused on four site level variables that potentially most influenced the occurrence of the studied taxa of arthropods (Table 3): (1) Canopy openness (an expression of light conditions) was measured under full foliage using a Sigma 4.5 mm F2.8 EX DC Circular FISHEYE HSM on July 15, 2010. Photographs were evaluated using a Gap Light Analyser 2.0 [24]. (2) Diameter at breast height of tree (an expression of tree diameter) was calculated from the circumference of a tree at 1.3 m from ground level. (3) Height of a tree (an expression of vertical biological frontier) was estimated and rounded in metres in the field. (4) Water distance (an expression of humidity) was calculated from the Euclidean distance of a tree to the River Becva.

**Table 3:** Results of descriptive statistics of study site level variables in a deciduous woodland area of Zebracka (sorted in alphabetical order).

Variable	Mean	S.E.	Minimum	Maximum
Canopy openness (%)	28.64	3.97	11.29	57.95
Diameter (cm)	82.29	6.35	49.68	135.35
Height (m)	13.73	0.50	10.00	15.00
Water distance (m)	104.87	18.47	3.00	230.00

### Statistical Analyses

For identification of sufficient number of trapping occasions, sample-based rarefaction with 95% confidence intervals computed using a Mao Tau function [2] and the Chao estimation function [25] were used. Analyses were computed in EstimateS 8.2 [26]. The number of randomisations was set at 1,000, with strong hash encryption and randomisation of samples without replacement. The upper abundance limit for rare or infrequent species was set at 10. I used the classic formulae for Chao for bias correction [26].

Correlation of taxa between seasons was evaluated using Spearman correlation coefficient in R.

Spatial autocorrelation was preliminary tested by randomized Geary’s C test using packages spdep and RANN in R [27].

For the final analyses with and without space, I used multivariate statistical methods provided by CANOCO for Windows version 4.5 [28]. All species data were square-root transformed, as is recommended for trapping designs [29]. The length of gradient in detrended correspondence analysis (DCA) in each season was at <2, which demonstrated that the data did not reveal high heterogeneity [28]. I therefore used redundancy analysis (RDA), a constrained linear ordination method [29]. I used focused scaling on inter-species correlations, and species scores divided by S.D., Monte-Carlo permutation tests with significance of canonical axes together (9,999 permutations) under the full model were used [29].

During the process of variance partitioning [30], two sets of environmental categories were used. Each fraction was measured based on three multivariate analyses using combinations of taxa, variables and co-variables [9], [31]. The first set of explanatory variables was composed of five most commonly spatial variables [32], namely geographical coordinates of the sampling points (x, y) and their squares and cross-product terms (x^2^, y^2^, xy) [8]. The cubic terms [30] were not included, keeping a comparable number of factors for the three explanatory data sets [8]. Thus, I used firstly space (x, y, xy, x^2^ and y^2^) as variables and habitat (Canopy openness, Diameter, Height and Water distance) as co-variables, then the same in reverse order, and finally with all variables included [9]. I visualised the results of variance partitioning using a two-circle Venn diagram.

The shared explained variance and *p* values of habitat variables were employed with a Monte Carlo permutation test (9,999 permutations) under the full model [29]. For the resulting ordination diagrams, I used RDA environmental ordination plots created in CanoDraw 4.14 [28].

## Results

The number of trapped taxonomical groups (Table 2) was 23 (7,318 individuals) in 2009 and 14 (32,326 individuals) in 2010. Total number of taxa was 24 (39,644 individuals), while 13 taxa overlapped in both seasons (Table 2; 4).

### Taxa Accumulations and Correlations in Distribution between Seasons

**Figure 2:**
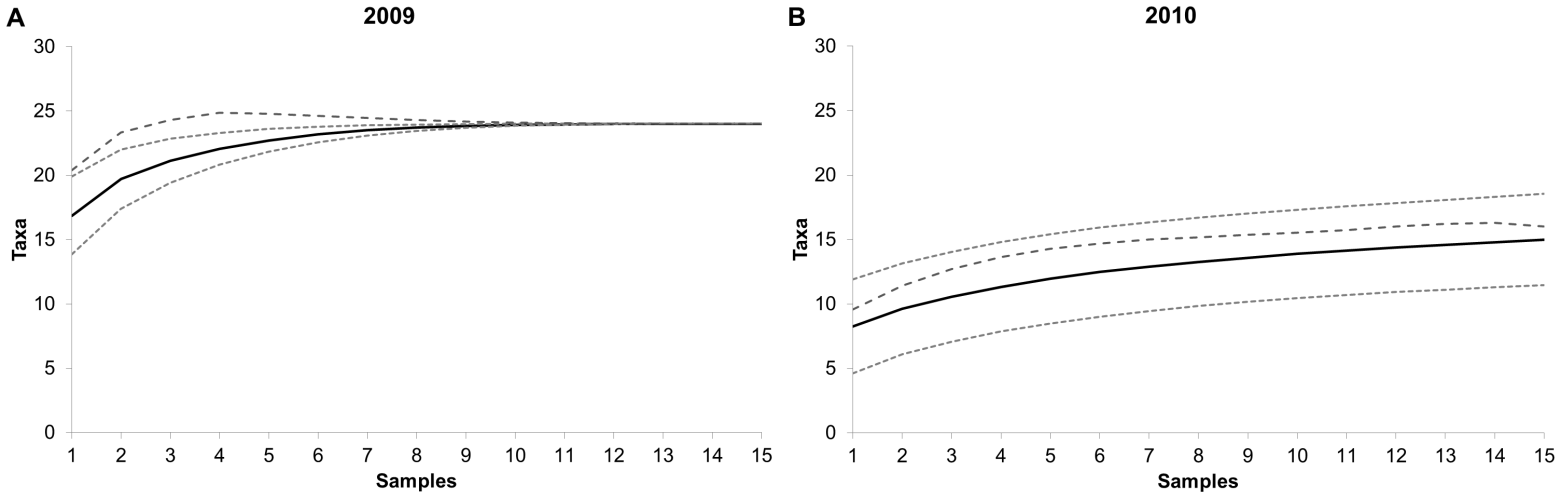
Taxa rarefactions and estimates of total richness of the trapped arthropods in the deciduous woodland. Complete data for taxa from all samples are included from a) 2009 and b) 2010. The solid black line shows a sample-based rarefaction of assemblages; the two surrounding light-grey dashed lines are Mao Tau estimates with 95% confidence intervals and the dark-grey dashed line is the Chao 1 estimate of the total number of taxa.

Sample-based rarefactions were made separately for each season (Fig. 2) and indicated that trapping success was higher for window traps than for panel traps. The curve reached its asymptote in 2009 (Fig. 2a), but not in 2010. However, the Chao estimate seemed to approach the total number of taxa (Fig. 2b), suggesting that the majority of the taxa in the study area were represented in the forthcoming analysis and that the number of samples was sufficient. Except of millipedes from order Julida, there was no correlation structure between 13 taxa overlapped in both seasons (Table 4).

**Table 4:** Correlation of taxa distribution in traps between the study seasons 2009 and 2010 in a deciduous woodland area of Zebracka (sorted in alphabetical order).

Taxa	*r*	*p*
Araneae	−0.05	0.87
Coleoptera	0.10	0.72
Dermaptera	−0.17	0.55
Diptera	0.21	0.46
Ephemeroptera	−0.14	0.64
Hemiptera	0.12	0.67
Hymenoptera	0.27	0.34
Julida	0.89	<0.0001
Lepidoptera	0.41	0.13
Mecoptera	0.38	0.16
Opilionida	−0.23	0.25
Pseudoscorpionida	−0.15	0.58
Trichoptera	−0.34	0.21

### Preliminary Tests on Spatial Autocorrelation of Taxa Richness and Habitat Variables

Richness of study taxa was not spatially autocorrelated in either year (Geary’s C_2009_ = 0.78, *p = *0.19; Geary’s C_2010_ = 0.94, *p = *0.41), with the same being the case for Diameter (Geary’s C = 0.67, *p = *0.10) and Height of tree (Geary’s C = 0.63, *p = *0.08). On the other hand, two site level variables were spatially autocorrelated: Canopy openness (Geary’s C = 0.34, *p = *0.0060) and Water distance (Geary’s C = 0.13, *p = *0.0005).

### Response of Arthropod Communities using Multivariate Statistics

**Figure 3:**
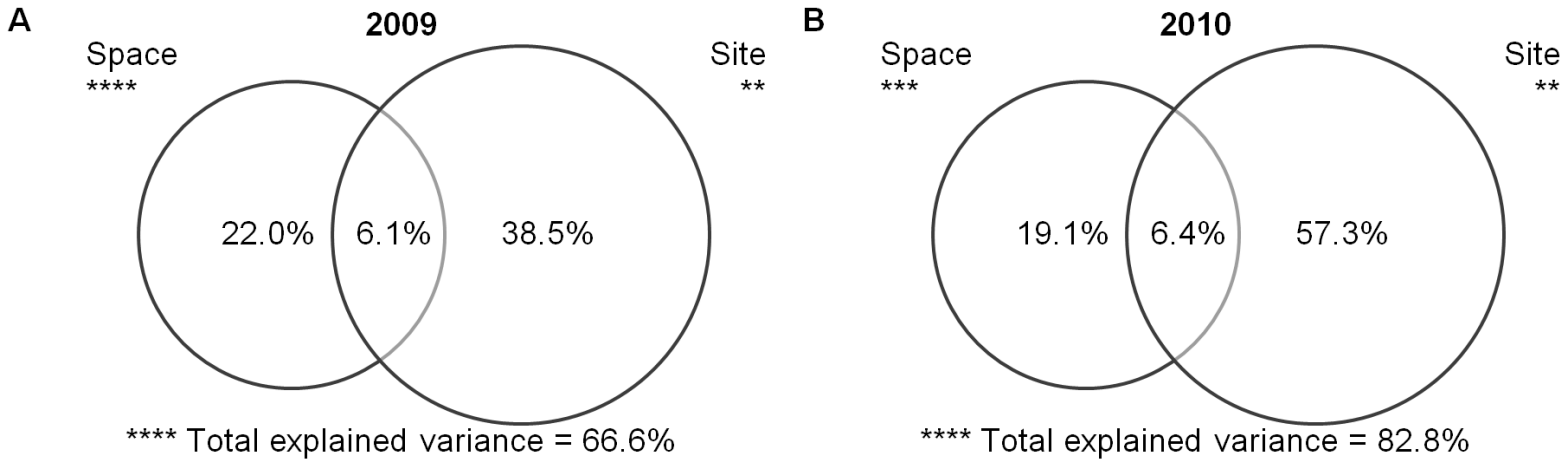
Venn diagrams. Figures are demonstrating percentage values of total, shared (values in circle overlaps) and independent explained variance of space and site using variance partitioning in a) 2009 and b) 2010 in a deciduous woodland area of Zebracka (**p<0.01; ***p<0.001; ****p = 0.0001).

Venn diagrams (Fig. 3) demonstrate that the total explained variance was high in both seasons. Site level variables explained a high level of variance (especially in 2010) and the percentage of shared variance did not much differ between study seasons. In contrast, the significance of space was high in both seasons. This indicated potential bias in the response of arthropods to site level variables in analyses that do not include space.

**Figure 4:**
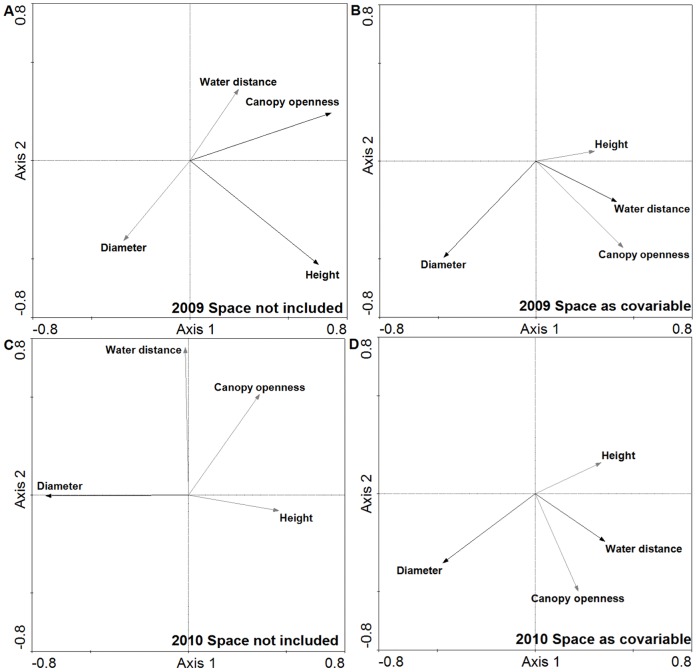
Relative position of environmental site level variables in RDA environmental ordination plots. Figures are with respect to taxa composition in a) 2009 without spatial covariables, b) 2009 with spatial covariables, c) 2010 without spatial covariables, and d) 2010 with spatial covariables, all in a deciduous woodland area of Zebracka (habitat variables *p*<0.05, black; *p* = n.s., grey).

The main difference was that environmental site level variables were in all cases differently rotated along the first axis. It can be seen that Diameter is separated from other variables on the second axis, and that Height has a different influence than Canopy openness and Water distance, both of which have nearly the same effect on taxa composition (Fig. 4).

The analyses showed that the level of total explained variance was lower when the effect of space was included in the analyses. On the other hand, the significance of the results at the site level increased (Table 5). Furthermore, the values of shared variance and significance differed strikingly between analyses with and without spatial variables (Table 5) and the relative position of habitat variables in RDA was highly variable (Fig. 4). Including space led to more accurate results, because Water distance and Diameter were significant, showing approximately the same ratio of variance and direction in both seasons (Table 5). The results without spatial co-variables were much more disordered (e.g. the effect of Diameter) and were also relatively difficult to explain (Table 5; Fig. 4).

**Table 5:** Results of taxa composition response to study site level variables in a deciduous woodland area of Zebracka, with space included as co-variable (left) and not included (right).

Variable	Trace	F	*p*	Variable	Trace	F	*p*
Space as co-variable				Space notincluded			
*2009*							
Total	0.385	2.72	0.0024	Total	0.446	2.01	0.0118
Water distance	0.234	5.88	0.0001	Canopyopenness	0.180	2.85	0.0265
Diameter	0.135	2.72	0.0287	Height	0.169	2.65	0.0331
Canopyopenness	0.100	1.87	0.12	Water distance	0.061	0.84	0.46
Height	0.073	1.30	0.25	Diameter	0.059	0.82	0.50
*2010*							
Total	0.573	6.66	0.0013	Total	0.636	4.38	0.0033
Water distance	0.310	7.49	0.0027	Diameter	0.323	6.21	0.0100
Diameter	0.217	4.27	0.0358	Height	0.138	2.08	0.15
Height	0.128	2.15	0.14	Canopyopenness	0.089	1.28	0.28
Canopyopenness	0.045	0.66	0.50	Water distance	0.018	0.24	0.76

Variables are sorted by value of trace (from highest to lowest).

## Discussion

As arthropods dominate the landscape with respect to their abundance and richness, local trapping success may be highly dependent on habitat and landscape heterogeneity [33]. Much of the current research uses mass trapping designs, not only for eradication of potential pests, but also for estimating biodiversity [34], [35], [36]. It is known, but often overlooked, that a large number of samples does not necessarily imply more significant results [37], [38], [39]. From this point of view, appropriate statistical design [9], [29], [31], [40] may lead to a higher significance without major exploitation of the environment, which is illustrated on rarefaction curves therein.

The trapping methods used in this study are mainly used for flying arthropods [41], [42]. However, when traps are suitably designed, they are known also to trap flightless fauna [18]. This is illustrated by sedentary taxa such as snails (Gastropoda) [43] or pillbugs (Isopoda) in this study.

Økland [44] writes that the total explained variance in ordination methods using variance partitioning, even using sets of carefully selected explanatory variables, is normally 20–50%, and occasionally up to 65%. On the other hand, inclusion of the spatial autocorrelation [9], [45] into analyses often leads to lowering of the explained variance of habitat variables [46], as in this study.

Previous studies, similar to this case, have used multivariate analysis with variation partitioning to separate the confounding effects of space and environment [8], [47], [48]. The results show that including spatial autocorrelation in analyses may lead to a more accurate outcome while its exclusion may lead to wrong conclusions [10], [17]. Even if there were differences in trapped arthropods between seasons, the response to habitat variables was, surprisingly, nearly the same in both seasons.

The results showed that it is necessary, for the study of organismal distribution spatial patterns using site level variables, to take spatial autocorrelation into account [46], [47], [48]. This also makes it possible to distinguish when the spatial structure is mainly due to biotic interactions with an underlying unmeasured environmental factor, or a common spatial gradient shared by data on taxa and environmental variables [8], [30]. Results of variance partitioning showed that spatial terms gave a lesser explanation of variance than did site, although significance of space and shared variation led to a much better explanation than when spatial autocorrelation was not included [49].

In the context of this article, there is a strong need for spatial autocorrelation to be included in the analysis, as it is also in similar relatively small-scale studies.

Distance to the River Becva and Diameter of tree were significant habitat variables, when spatial autocorrelation was included. Water distance reflected the humidity of habitats, which is known to influence arthropod communities [50], [51], Diameter is a traditional habitat variable in studies, used to explain diameter and age of the tree, especially for saproxylic communities [4], [52] and thus the response of communities to Diameter was not surprising.

## Conclusions

This study showed that neglecting spatial autocorrelation could possibly lead to wrong conclusions in small site level studies and, furthermore, that inclusion of spatial terms may lead to more accurate and less ambiguous outcomes. Lower sampling intensity, when appropriately designed, is able to gain sufficient results and may lead to a higher significance without major exploitation of the environment.
